# Artificial intelligence in animal breeding and genetics: applications, opportunities, and challenges

**DOI:** 10.1093/af/vfaf058

**Published:** 2026-01-08

**Authors:** Gota Morota, Hyo-Jun Lee, Seung-Hwan Lee, Hee-Bok Park, Akio Onogi

**Affiliations:** Department of Artificial Intelligence Convergence, Kangwon National University, Chuncheon, 24341, Republic of Korea; Division of Animal and Dairy Science, Chungnam National University, Daejeon, 34134, Republic of Korea; Department of Animal Resources Science, College of Industrial Sciences, Kongju National University, Yesan, 32439, Republic of Korea; Department of Life Sciences, Faculty of Agriculture, Ryukoku University, Otsu, Shiga 520-2194, Japan

**Keywords:** animal breeding, deep learning, neural networks, pheno­mics, prediction, quantitative genetics, selection

Implications

- Applications of artificial intelligence in animal breeding and genetics can be broadly categorized into two areas: phenotype generation and predictive genetic modeling.
- Previous studies have primarily focused on using artificial intelligence for digital phenotyping, whereas its integration with genomic prediction remains in its early stages.
- Artificial intelligence, in the form of foundation models, is becoming an indispensable tool for generating animal phenotypes from image and sensor data, with highly promising future prospects.
- The application of artificial intelligence to tabular data, such as single nucleotide polymorphism datasets, continues to pose significant challenges in the context of genomic prediction.

## Introduction

Animal science has become a data-rich field, particularly within the area of animal breeding and genetics, where millions of phenotyped and genotyped animals are now available across livestock species databases. Genomic selection is now routinely used for genetic evaluations in many livestock species. Meanwhile, artificial intelligence (AI) has become a major focus in recent years, appearing in nearly every aspect of our daily lives. Animal breeding and genetics is no exception, and questions remain regarding where and how AI can be effectively integrated into this field. Broadly speaking, the role of AI in animal breeding and genetics is twofold: phenotype generation and predictive genetic modeling. Although the utility of AI in animal breeding and genetics has been reviewed previously, most existing studies primarily highlight its applications in digital phenotyping (Morota et al., 2018; Koltes et al., 2019; Grohmann and Decker, 2024; Klingström et al., 2025). This is because, by harnessing digital data sources, such as images, video recordings, and sensor signals, digital phenotyping enables efficient prediction and classification of animal phenotypes. However, the convergence of AI and genomic prediction models is still in its early stages. In light of this, this feature article emphasizes not only the application of AI in animal phenotyping but also the exploration of phenotype–genotype relationships through the lens of AI.

Although strictly speaking, generalized linear mixed models, machine learning, and deep learning (DL), all fall under the umbrella of AI, the term AI is used throughout this paper to specifically refer to DL. Deep learning is one of the fastest-growing topics in animal science, likely due to the increasing democratization of DL technologies. For example, the availability of affordable, high-performance graphics processing units, the widespread use of DL software frameworks such as Keras and PyTorch in both Python and R, and the growing number of DL textbooks and online tutorials have all greatly contributed to making DL more accessible to researchers across disciplines. Arguably, while many animal breeders and geneticists believe that the era of AI is arriving in their field, not everyone is fully aware of the current status of AI applications in breeding and genetics. Moreover, there is still no consensus on the specific areas in which AI truly excels, especially given that the mixed model equations developed by Henderson, which simultaneously derive best linear unbiased estimators and best linear unbiased predictors (Henderson et al., 1959; Henderson, 1963), remain a powerful and widely used approach in genetic evaluations of animals. Therefore, this feature article highlights not only the successes and challenges associated with applying AI to animal breeding and genetics based on the current literature but also outlines potential future research directions in this field.

## Artificial Intelligence for Phenomics

One of the areas where the presence of AI has risen at a commensurate rate is in phenotyping animals using AI-backed precision livestock farming technologies, or phenomics. In particular, digital phenotyping is relevant for animal geneticists; however, when phenotyping is extended continuously over time, it can also be regarded as monitoring, which has important implications for animal management. When combined with high-throughput genotyping or sequencing technologies, the use of AI for phenotyping holds great promise as a means to more efficiently link animal phenotypes with pedigree and genomic information, such as single nucleotide polymorphisms (SNPs), to drive genetic improvement. This is because the rate of genetic gain is closely tied to the quantity and quality of phenotypic data collected. Improved accuracy of phenotypes leads to greater genetic gain for target traits and breeding objectives. In simpler terms, “in the age of the genotype, phenotype is king” (Coffey, 2020).

Two major technologies driving AI-based phenomics are wearable sensors and computer vision systems, each offering distinct advantages and challenges. Wearable sensors excel at capturing individual-level activity and behavioral data, and they can also enable animal identification when integrated with radio-frequency identification technology. However, their widespread use is often limited by the high cost of deploying sensors for every animal and the practical difficulties associated with maintaining sensor attachments over time. In contrast, computer vision systems provide a scalable, non-contact approach, capable of simultaneously phenotyping multiple animals using a single camera. Yet, one of their primary challenges lies in accurately identifying individual animals, particularly over extended periods of time.

In this paper, we focus on the use of computer vision, as this technology leverages the power of AI to both operate cameras and process image data. In contrast, successful applications of AI in wearable sensor technologies remain relatively limited. This is largely because DL does not consistently excel at processing wearable sensor data, such as tri-axial accelerometer signals, which are tabular in nature and may not fully exploit the representational advantages of DL architectures. This point is discussed further in a later section.

**Figure 1.**
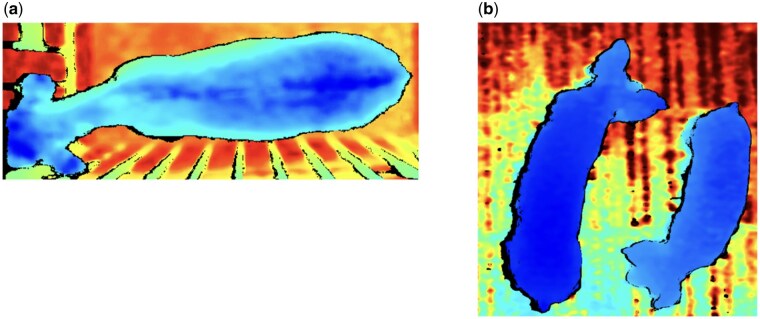
Example of depth image data of (a) cattle and (b) pigs. The color variation in each depth image corresponds to the distance between the camera and the object.

As stated earlier, the application of AI in animal breeding and genetics has been most widely reported in phenotyping using computer vision technologies. The types of cameras employed vary widely, including surveillance cameras, industrial RGB cameras, thermal cameras, and depth cameras. An example of depth image data is shown in Figure 1. The most common phenotyping setup involves mounting a camera near the ceiling to capture top-down images. This configuration keeps the camera out of the animals’ reach and reduces the likelihood of occlusion. Broadly speaking, computer vision is used for two tasks: phenotyping morphometric characters and animal activity. The former includes phenotyping body weight and body condition score to track animal growth dynamics or health conditions. The latter includes monitoring feeding, movement, posture, and social interactions.

## Computer Vision for Morphological Characters

In DL, every layer performs a specific transformation that helps the model learn progressively richer features needed for prediction or classification. A transformation is the mathematical operation a layer applies to its input, and a layer is a structural unit that performs one such operation. The first layers identify basic patterns, such as edges and corners, while subsequent layers merge these into more complex forms, enabling the detection of meaningful parts of objects. Early applications of DL for phenotyping morphological characters include the use of DL for generating segmented animal images using Mask R-CNN or YOLO (Bi et al., 2023). The segmented images were then used to extract biometric descriptors, such as length, width, height, and volume, with occasional manual feature engineering. This step is followed by fitting a regression or classification model using those biometric descriptors as predictors. This method is therefore considered a two-step approach, where image segmentation is performed using DL and then estimated biometric descriptors are used for prediction. When Mask R-CNN was combined with a linear mixed model for longitudinal dairy cow body weight prediction using depth image data, the study reported coefficients of determination of 0.90 and a mean absolute percentage error of 4.70% according to a leave-cows-out cross-validation scheme (Bi et al., 2023).

**Figure 2.**
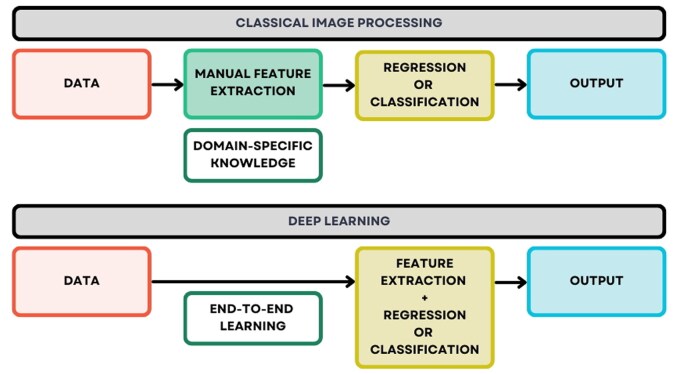
Comparison between classical image processing and deep learning. End-to-end learning in deep learning refers to training a model to perform an entire task directly from raw input to final output, eliminating the need for manually designed intermediate stages or handcrafted feature extraction.

A recent trend is the application of DL that enables end-to-end learning. Here, end-to-end learning refers to training a model to perform an entire task directly from raw input to final output, without the need for manually engineered intermediate steps or handcrafted feature extraction (Figure 2). These end-to-end DL approaches eliminate the need for tedious image preprocessing steps, thereby streamlining the workflow and reducing human intervention. In other words, once animal image data is fed into a DL model, it can directly produce prediction or classification outputs. For example, MobileViT, a lightweight hybrid architecture that combines convolutional neural networks (CNNs) and vision transformers, achieved a mean absolute percentage error of 3–4% when predicting the body weight of unobserved pigs using depth image data (Bi et al., 2025). Similarly, an end-to-end Vision Transformer model developed to estimate body condition score in dairy cows from RGB and depth images achieved accuracies ranging from 64% to 92%, highlighting the importance of dataset splitting strategies, dataset size, sample uniqueness, and experimental design (Winkler et al., 2024).

## Computer Vision for Activity and Behavior

Applications of DL are also making inroads into the estimation of animal activity and behavior. A ResNet-inspired CNN was used to estimate individual feed intake, in combination with Faster R-CNN for cow identification via collar-mounted number plates in open cowshed conditions (Bezen et al., 2020). The results showed a mean absolute error of 0.127 kg for feed intake estimation and an identification accuracy of 93.65%. Another example is the phenotyping of social and aggressive interactions in pigs, which are critical for ensuring animal welfare, reducing injuries and stress, optimizing productivity, and enabling precision management. A combined approach using VGG16, a type of CNN, and long short-term memory, a type of recurrent neural network, was applied to automatically recognize aggressive behaviors in pigs (Chen et al., 2020). The model achieved an accuracy of 97.2%, which further improved to 98.4% when every other frame was omitted, effectively reducing computation time by half. These DL-based phenotyping technologies play a critical role in supporting the application of social genetic effect models, also known as indirect genetic effect models, which allow breeders to select animals that are not only individually superior but also socially beneficial. Such selection strategies can lead to improved group performance, enhanced animal welfare, and greater sustainability in livestock production.

Recent climate change further underscores the need to breed animals that are robust to heat stress. An end-to-end computer vision model based on the VideoMAE Transformer was developed to continuously estimate the respiratory rate in dairy cows using RGB video data (Wang et al., 2024). By capturing periodic movements around the abdominal region, particularly the oscillations of the cow’s flank, indicative of respiratory activity, the model achieved a mean absolute error of 2.58 breaths per minute, a root mean squared error of 3.52 breaths per minute, a root mean squared prediction error of 15.03%, and a Pearson correlation coefficient of 0.86. Together, these advances demonstrate the growing power of DL-based computer vision to deliver accurate assessments of animal activity and behavior that are essential for welfare monitoring and data-driven breeding.

## Computer Vision for Carcass Phenotypes

The image data analyzed in the aforementioned examples were all derived from live animal phenotyping systems, which are the primary focus of this feature article. However, the use of DL for carcass phenotypes is also on the rise. For instance, BTENet is a DL framework developed for the automated estimation of pig backfat thickness in a carcass grading system, using head-side images (Lee et al., 2022). The framework integrates a segmentation module (U-Net style) and a thickness estimation module (ResNet50) to jointly identify the backfat region and predict its thickness. In addition, the authors constructed a newly curated dataset, PigBT, comprising annotated pig carcass images with backfat masks and thickness labels. On the test set, BTENet achieved a Pearson correlation of 0.92, a mean absolute error of 1.28 mm, and a mean absolute percentage error of approximately 6.4%, demonstrating strong predictive performance. Overall, these advances show that computer vision is a powerful tool for generating carcass phenotypes for genetic improvement.

## Future Perspectives

One additional challenge that remains is image data annotation. In cases when there is a large pool of unlabeled images but limited labeled data, self-training or pseudo-labeling represents a viable semi-supervised learning strategy. In self-training, a model is first trained on a small labeled dataset and then uses its own predictions to assign labels to unlabeled data. These pseudo-labels are treated as ground truth for a second round of training, allowing the model to learn from both labeled and pseudo-labeled samples. For example, self-training can be used to efficiently expand labeled cow segmentation datasets by training deep learning segmentation models with only a small number of manually annotated images, which are then used to automatically generate pseudo-labels for thousands of unlabeled frames. These pseudo-labels are subsequently refined manually, turning weak auto-labels into reliable ground truth, enabling iterative retraining and rapid dataset scaling while minimizing manual annotation time (Liao et al., 2025).

Another approach that holds promise for computer vision–based animal phenomics is the use of zero-shot learning. Zero-shot learning enables models to recognize unseen classes or perform new tasks without explicit training data by leveraging semantic knowledge. This approach is particularly valuable in animal science, where annotated training data are often scarce. Even when large volumes of image data are collected, access to computational resources for extensive labeling and training may be limited. Zero-shot learning has the potential to bridge the gap between data-rich and data-poor domains, allowing animal computer vision systems to scale without exhaustive manual labeling, generalize across breeds and behaviors, and adapt to new conditions by exploiting semantic information or pretrained multimodal models. Attempts to apply zero-shot learning in animal computer vision have only recently begun, with the availability of zero-shot learning tools, such as the Segment Anything Model family. For example, the zero-shot segmentation performance of the Segment Anything Model was evaluated for chicken segmentation tasks, achieving a mean intersection over union of 94.8%, and demonstrating its potential for poultry tracking when combined with a multi-object tracker (Yang et al., 2024). In addition, a modified Segment Anything Model was used to automatically segment and evaluate the thermal conditions of individual chickens, achieving an intersection over union of 85.5%, a recall of 91.0%, and an F1-score of 92.3% (Saeidifar et al., 2024). Similarly, a variant of the Segment Anything Model can be used for constructing high-quality computer vision datasets from raw livestock surveillance videos by combining zero-shot referring-and-grounding models with data distillation mechanisms. This data augmentation process can lower the barrier to building benchmark datasets in precision livestock farming and can accelerate the deployment of computer vision applications (Wu et al., 2024). It is likely that the future direction of this line of research will involve developing large-scale, general-purpose AI systems trained on diverse datasets and capable of adapting to multiple downstream tasks, forming the foundation for computer vision applications in animal science. Such systems are referred to as foundation models (Li et al., 2024).

A common misconception is that all camera-based phenotyping qualifies as high-throughput, which is not always the case. In plant breeding and genetics, the term high-throughput phenotyping is often used interchangeably with image-based phenotyping because drone- or greenhouse-based imaging systems can generate large volumes of data from sessile plants. In contrast, some animal computer vision studies cannot yet be classified as high-throughput, as substantial manual labor is still required to operate cameras and capture usable images due to animal movements. Computer vision–based phenotyping can generally be divided into two components: system setup (hardware) and image processing and analysis (software). For camera-based phenotyping to be considered high-throughput, the system must be semi- or fully automated (Kadlec et al., 2022), enabling large-scale, consistent data acquisition with minimal human intervention. High-throughput phenotyping implies the ability to capture significantly more data than is possible through manual phenotyping. Conversely, when image or video collection involves manual procedures or control, the term digital phenotyping or image-based phenotyping is more appropriate.

## Genetic Analysis of Image-Derived Phenotypes

Image data obtained from computer vision can be used to predict phenotypes that serve as traits in genetic analyses. Image-based phenotyping holds great promise for accelerating genetic improvement while reducing phenotyping costs. A broad integration of these technologies into breeding pipelines is anticipated in the coming years, establishing it as a leading phenotyping approach. Such image-based approaches may enhance consistency and precision while lowering phenotyping costs for traits that are already routinely measured. Automatically collected image-based phenotypes can also facilitate large-scale phenotyping of hard-to-measure traits that are otherwise costly or labor-intensive to record. Furthermore, computer vision can be used to generate auxiliary traits that are correlated with primary target traits to enhance genomic selection. Genetic analyses based on pedigree or genomic information are increasingly being applied to computer vision–derived traits related to growth or morphology (Gorssen et al., 2022; Manzanilla-Pech et al., 2023; Qin et al., 2024), behavior (Gorssen et al., 2022), and feed intake (Manzanilla-Pech et al., 2023). Regarding behavior, AI and machine learning technologies have also been applied to classify animal behaviors using accelerometer sensor outputs (Alghamdi et al., 2022; Riaboff et al., 2022). Furthermore, genetic analyses of traits derived from wearable sensor data are gaining momentum (Onogi et al., 2024).

## Artificial Intelligence for Bridging the Phenome to Genome

**Figure 3.**
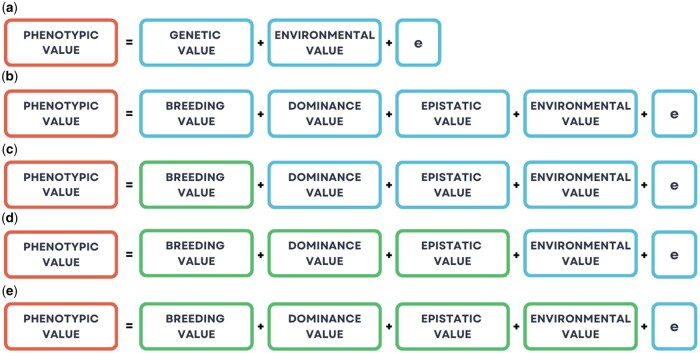
Phenotypic value can be decomposed into genetic value, macro-environmental value, and a residual term, which captures the effects of micro-environmental variation and phenotyping error (a). The genetic value itself can be further partitioned into additive (breeding), dominance, and epistatic components (b). Because artificial intelligence, particularly deep learning, excels at capturing nonlinear relationships, its potential usefulness may lie in predicting genetic values (d) and phenotypes (e), which integrate both genetic and environmental components, rather than focusing solely on (c) breeding values.

The strength of AI in prediction lies in its ability to model nonlinear relationships between response and predictor variables. According to the theory of quantitative genetics, a phenotype can be partitioned into additive (breeding value), dominance, epistatic, environmental, and residual components. Genomic prediction is a statistical approach used to predict breeding values or genetic values in animals, encompassing all forms of gene action, including additive, dominance, and epistatic effects. Genomic selection, on the other hand, is a breeding strategy or decision-making process that uses these predicted values to select individuals for mating, culling, or advancement within a breeding program. Past studies have shown that when a trait is largely additive in nature, AI models do not necessarily outperform linear models, such as genomic best linear unbiased prediction (GBLUP), which remains the standard choice in animal breeding and genetics. Thus, although the prediction of breeding values is central to many animal breeding programs aiming to achieve genetic improvement, this is not necessarily the area in which AI excels. For this reason, the potential usefulness of AI may lie in predicting 1) genetic values and 2) phenotypes per se, which integrate both genetic and environmental components, with enviromics serving as a means to capture the latter information (Figure 3). Practical examples include predicting both additive and dominance effects to inform mating design selection or predicting phenotypes directly to support management and marketing decisions. Furthermore, because the GBLUP framework can be readily extended to accommodate dominance, epistasis, and environmental effects (Onogi et al., 2021), AI must demonstrate clear superiority over the GBLUP framework to justify its adoption. Although it is outside the scope of the current paper, AI can be used to predict causal, regulatory, or functional variants in farm animals. However, this remains a relatively small and emerging area compared with humans.

## Real Data Examples in Genomic Prediction

Because simulation studies are often designed in ways that favor AI performance, this feature article focuses on studies that used real animal data. Moreover, there is currently no literature that has attempted to predict phenotypes jointly incorporating both genetic and environmental components using DL; instead, existing real-data studies have exclusively targeted the prediction of genetic values. It is also common practice to perform pre-selection of SNPs or apply dimensionality reduction techniques to reduce the number of SNPs fed into DL models, thereby lowering computational demands.

Early applications of neural networks for genomic prediction focused on shallow architectures, typically consisting of feed-forward neural networks (i.e., multilayer perceptrons) with a single hidden layer and only a few neurons (Gianola et al., 2011). This pioneering study on milk production in Jersey cows spurred further investigations into the potential of deeper network architectures for improving predictive performance in genomic prediction. To date, comparisons with baseline linear models, such as GBLUP, have yielded somewhat mixed results. For example, CNNs have shown better predictive performance than linear models for the lifetime number of litters and lifetime pig production (Hong et al., 2024). A multilayer perceptron with up to four hidden layers exhibited the lowest mean squared error of prediction, but no difference in prediction correlation was observed compared with linear models for broiler body weight (Passafaro et al., 2020). Moreover, the predictive performance of DL has not always been proportional to the size of the training dataset, indicating that simply increasing the amount of data does not automatically lead to improved prediction accuracy.

In addition, the integration of DL and GBLUP into a single framework has been proposed. In this context, when applied to carcass weight, eye-muscle area, backfat thickness, and marbling score in Korean native cattle, the deepGBLUP model achieved higher predictive accuracy than its linear model counterparts (Lee et al., 2023). In a similar attempt, the DLGBLUP model, a hybrid approach combining DL and GBLUP, yielded lower mean squared errors but showed no improvement in prediction accuracy for a wide range of traits, including milk yield, fat yield, protein content, somatic cell score, conception rate, and depth of furrow in Holstein dairy cattle (Shokor et al., 2025).

On the other hand, CNNs yielded lower predictive ability than GBLUP for sire conception rate records in U.S. Holstein bulls (Abdollahi-Arpanahi et al., 2020). Similarly, feed-forward neural networks with up to four layers performed consistently no better than GBLUP for six quantitative traits, including off-test body weight, off-test back fat thickness, off-test loin muscle depth, number of piglets born alive, number of piglets born dead, and number of piglets weaned in pigs (Wang et al., 2025). In a composite beef population, GBLUP achieved the highest prediction accuracy for birth weight and yearling weight, as well as the lowest mean squared error, outperforming CNNs (Hay, 2024).

## Challenges of Deep Learning to Analyze Tabular Data

The overall consensus from previous genomic prediction studies is that DL does not consistently outperform its linear model counterparts, and when it does, the improvements are often marginal. There is also a tendency for DL to yield better predictive performance when evaluated using the mean squared error. The mean squared error emphasizes absolute differences between predicted and observed values, whereas predictive correlation or prediction accuracy focuses on rank differences, which are generally more relevant for the selection of candidate animals in breeding programs. This raises an important question: why has DL achieved remarkable success with text and image data, yet its superiority with tabular data, such as genomic datasets, remains unclear?

Recent theoretical studies have attempted to explain this phenomenon observed in tabular data, particularly in the context of DL (Gorishniy et al., 2021; Grinsztajn et al., 2022). First, DL generally performs best when inputs are continuous and normalized, such as pixel intensities in images or word embeddings in text. However, DL models tend to struggle with categorical inputs, as is the case with SNPs, which typically take only three discrete values representing the number of reference alleles. Second, although DL models tend to assume smooth and continuous relationships between SNPs and phenotypes, genetic architectures are often highly discontinuous, with many SNPs having no effect while a few exerting large effects. Such genotype–phenotype mappings exhibit irregular and heterogeneous patterns, a characteristic sometimes described as irregular data in tabular learning research. Third, DL models may lack robustness to uninformative or noisy features that do not contribute to phenotypic variation, thereby introducing noise and reducing generalization ability, because dense connections propagate signals from all inputs. In general, DL models lack built-in feature selection mechanisms and must rely on gradient updates and regularization to suppress uninformative features. This issue is particularly relevant in genomic prediction, where the vast majority of SNPs are weakly informative or entirely uninformative. In addition, DL for genomic prediction is reported to not learn SNP markers or epistatic effects but instead rely on genetic relatedness, engaging in shortcut learning, in which models exploit simple correlations rather than the features they are intended to learn (Ubbens et al., 2021). This is supported by the finding that a DL model given only the locations of matches between markers for pairs of individuals performs as well as one using full marker data. These potential explanations further motivate the development of DL approaches specifically tailored to tabular data. An alternative avenue to leverage the strengths of DL is to convert tabular data into image representations; however, this approach still requires further investigation (Bi et al., 2025).

## Conclusions

Artificial intelligence, particularly DL, is reshaping animal breeding and genetics through advances in phenotyping and computer vision. Emerging techniques, such as self-training, zero-shot learning, and foundation models, hold promise for reducing data-annotation demands and improving transferability across populations and environments, advancing AI as a cornerstone of sustainable, data-driven phenotyping. When combined with genomic information, AI offers powerful tools for accelerating genetic gain, improving animal welfare, and enhancing decision-making. However, DL methods have not yet consistently outperformed established statistical approaches, such as GBLUP, in genomic prediction. Future progress will depend on developing DL architectures specifically designed for tabular data, such as SNP data, and on integrating AI with quantitative genetic theory to better capture complex genotype–phenotype relationships.

## Author Biographies

**Gota Morota** is an Associate Professor in the Department of Agricultural and Environmental Biology at the University of Tokyo, where he works on quantitative genetics and phenomics with applications in animal and plant breeding. His research focuses on the development and application of statistical approaches for the inference and prediction of complex traits in agricultural species using high-dimensional omic data. He is also interested in applying digital phenotyping technologies to generate novel or difficult-to-measure phenotypes for genetic analysis. Morota began his career as an Assistant Professor in the Department of Animal Science at the University of Nebraska–Lincoln before moving to Virginia Tech, where he served as an Assistant and later Associate Professor. He completed his BS in Agricultural Science at Obihiro University of Agriculture and Veterinary Medicine and earned his PhD in Quantitative Gene­tics from the University of Wisconsin–Madison.

**Hyo-Jun Lee** received his BS and MS degrees in Animal Science and his PhD in Bio-AI Convergence from Chungnam National University, South Korea. Following his doctoral studies, he worked as a postdoctoral research fellow at the Software Research Center of Chungnam National University. He is currently an Assistant Professor in the Department of Artificial Intelligence Convergence at Kangwon National University. His research interests include computer vision, deep learning, and bioinformatics.

**Seung-Hwan Lee** is a Professor in the Division of Animal & Dairy Science and the Department of Bio-AI Convergence at Chungnam National University, South Korea. He holds a PhD in Animal Breeding and Genetics from the University of New England, Australia. Dr Lee’s research pioneers the integration of artificial intelligence with livestock genetics to advance precision agriculture. His work focuses on developing deep learning algorithms for genomic prediction and computer vision systems for precision phenotyping, such as automated marbling scoring and back-fat estimation. By bridging biological big data with AI, he aims to optimize breeding programs and enhance the accuracy of meat quality evaluation. He is actively involved in the AI Convergence Research Center, supported by the Institute of Information and Communications Technology Planning and Evaluation grant funded by the Korean government, leading projects that apply machine learning techniques to solve complex challenges in animal breeding.

**Hee-Bok Park** is an Associate Professor in the Department of Animal Resources Science at Kongju National University, Republic of Korea, specializing in animal breeding and genetics. He earned his PhD in Quantitative Genetics and Genomics from Uppsala University, Sweden. His research focuses on genomic selection, identification of causal genes for economically important traits, and the application of big data analytics and artificial intelligence in animal genetics. Prior to joining Kongju National University, Dr Park served as a postdoctoral researcher at Lund University, Sweden, and held academic positions at Gyeongsang National University and Chungnam National University in the Republic of Korea. He also worked as a Research Associate at the National Institute of Animal Science, Rural Development Administration, Republic of Korea. His expertise spans advanced statistical methods for genetic analysis and genomic prediction in livestock populations, with particular emphasis on improving breeding programs through modern computational approaches.

**Akio Onogi** is a Professor in the Faculty of Agriculture at Ryukoku University. He holds a PhD in Agricultural Science from the University of Tokyo. He previously worked at the National Agriculture and Food Research Organization, where he researched crop breeding methods using statistics and machine learning, and at the Livestock Improvement Association of Japan, where he contributed to livestock breeding practice. His research interests include quantitative genetics and genomic prediction, statistical and computational methods for breeding, machine learning applications in crop and livestock improvement, and the integration of genomic, phenotypic, and environmental data to enhance selection accuracy and sustainability.
